# Bumblebees socially learn behaviour too complex to innovate alone

**DOI:** 10.1038/s41586-024-07126-4

**Published:** 2024-03-06

**Authors:** Alice D. Bridges, Amanda Royka, Tara Wilson, Charlotte Lockwood, Jasmin Richter, Mikko Juusola, Lars Chittka

**Affiliations:** 1 School of Biological and Behavioural Sciences, Queen Mary University of London, London, UK; 2 School of Biosciences, University of Sheffield, Sheffield, UK; 3 Neuroscience Institute, University of Sheffield, Sheffield, UK; 4 Present Address: Department of Psychology, Yale University, New Haven, CT USA

**Keywords:** Animal behaviour, Behavioural ecology

## Abstract

Culture refers to behaviours that are socially learned and persist within a population over time. Increasing evidence suggests that animal culture can, like human culture, be cumulative: characterized by sequential innovations that build on previous ones^1^. However, human cumulative culture involves behaviours so complex that they lie beyond the capacity of any individual to independently discover during their lifetime^1–3^. To our knowledge, no study has so far demonstrated this phenomenon in an invertebrate. Here we show that bumblebees can learn from trained demonstrator bees to open a novel two-step puzzle box to obtain food rewards, even though they fail to do so independently. Experimenters were unable to train demonstrator bees to perform the unrewarded first step without providing a temporary reward linked to this action, which was removed during later stages of training. However, a third of naive observer bees learned to open the two-step box from these demonstrators, without ever being rewarded after the first step. This suggests that social learning might permit the acquisition of behaviours too complex to ‘re-innovate’ through individual learning. Furthermore, naive bees failed to open the box despite extended exposure for up to 24 days. This finding challenges a common opinion in the field: that the capacity to socially learn behaviours that cannot be innovated through individual trial and error is unique to humans.

## Abstract (web summary)

Bumblebees can learn how to open a two-step puzzle box by observing another trained bee, indicating that these insects can use social learning to acquire a behaviour too complex to otherwise be learnt through individual trial and error.

## Main

Culture in animals can be broadly conceptualized as the sum of a population’s behavioural traditions, which, in turn, are defined as behaviours that are transmitted through social learning and that persist in a population over time^4^. Although culture was once thought to be exclusive to humans and a key explanation of our own evolutionary success, the existence of non-human cultures that change over time is no longer controversial. Changes in the songs of Savannah sparrows^5^ and humpback whales^6–8^ have been documented over decades. The sweet-potato-washing behaviour of Japanese macaques has also undergone several distinctive modifications since its inception at the hands of ‘Imo’, a juvenile female, in 1953^9^. Imo’s initial behaviour involved dipping a potato in a freshwater stream and wiping sand off with her spare hand, but within a decade it had evolved to include repeated washing in seawater in between bites rather than in fresh water, potentially to enhance the flavour of the potato. By the 1980s, a range of variations had appeared among macaques, including stealing already-washed potatoes from conspecifics, and digging new pools in secluded areas to wash potatoes without being seen by scroungers^9–11^. Likewise, the ‘wide’, ‘narrow’ and ‘stepped’ designs of pandanus tools, which are fashioned from torn leaves by New Caledonian crows and used to fish grubs from logs, seem to have diverged from a single point of origin^12^. In this manner, cultural evolution can result in both the accumulation of novel traditions, and the accumulation of modifications to these traditions in turn. However, the limitations of non-human cultural evolution remain a subject of debate.

It is clearly true that humans are a uniquely encultured species. Almost everything we do relies on knowledge or technology that has taken many generations to build. No one human being could possibly manage, within their own lifetime, to split the atom by themselves from scratch. They could not even conceive of doing so without centuries of accumulated scientific knowledge. The existence of this so-called cumulative culture was thought to rely on the ‘ratchet’ concept, whereby traditions are retained in a population with sufficient fidelity to allow improvements to accumulate^1–3^. This was argued to require so-called higher-order forms of social learning, such as imitative copying^13^ or teaching^14^, which have, in turn, been argued to be exclusive to humans (although, see a review of imitative copying in animals^15^ for potential examples). But if we strip the definition of cumulative culture back to its bare bones, for a behavioural tradition to be considered cumulative, it must fulfil a set of core requirements^1^. In short, a beneficial innovation or modification to a behaviour must be socially transmitted among individuals of a population. This process may then occur repeatedly, leading to sequential improvements or elaborations. According to these criteria, there is evidence that some animals are capable of forming a cumulative culture in certain contexts and circumstances^1,16,17^. For example, when pairs of pigeons were tasked with making repeated flights home from a novel location, they found more efficient routes more quickly when members of these pairs were progressively swapped out, when compared with pairs of fixed composition or solo individuals^16^. This was thought to be due to ‘innovations’ made by the new individuals, resulting in incremental improvements in route efficiency. However, the end state of the behaviour in this case could, in theory, have been arrived at by a single individual^1^. It remains unclear whether modifications can accumulate to the point at which the final behaviour is too complex for any individual to innovate itself, but can still be acquired by that same individual through social learning from a knowledgeable conspecific. This threshold, often including the stipulation that re-innovation must be impossible within an individual’s own lifetime, is argued by some to represent a fundamental difference between human and non-human cognition^3,13,18^.

**Fig. 1:**
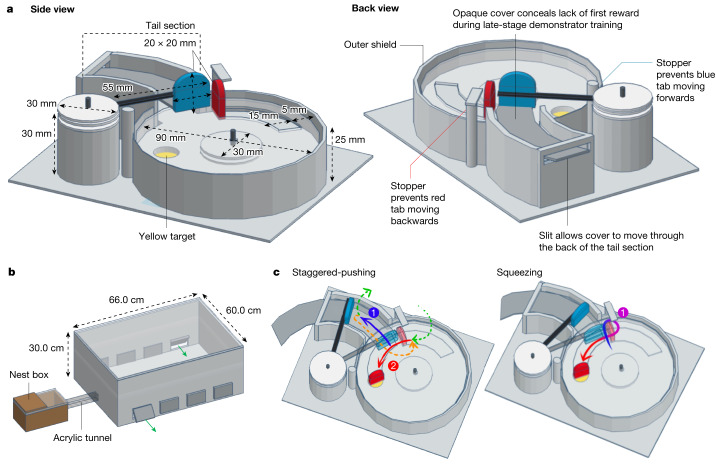
Two-step puzzle-box design and experimental set-up. **a**, Puzzle-box design. Box bases were 3D-printed to ensure consistency. The reward (50% w/w sucrose solution, placed on a yellow target) was inaccessible unless the red tab was pushed, rotating the lid anti-clockwise around a central axis, and the red tab could not move unless the blue tab was first pushed out of its path. See Supplementary Information for a full description of the box design elements. **b**, Experimental set-up. The flight arena was connected to the nest box with an acrylic tunnel, and flaps cut into the side allowed the removal and replacement of puzzle boxes during the experiment. The sides were lined with bristles to prevent bees escaping. **c**, Alternative action patterns for opening the box. The staggered-pushing technique is characterized by two distinct pushes (1, blue arrow and 2, red arrow), divided by either flying (green arrows) or walking in a loop around the inner side of the red tab (orange arrow). The squeezing technique is characterized by a single, unbroken movement, starting at the point at which the blue and red tabs meet and pushing through, squeezing between the outer side of the red tab and the outer shield, and making a tight turn to push against the red tab.

Bumblebees (*Bombus terrestris*) are social insects that have been shown to be capable of acquiring complex, non-natural behaviours through social learning in a laboratory setting, such as string-pulling^19^ and ball-rolling to gain rewards^20^. In the latter case, they were even able to improve on the behaviour of their original demonstrator. More recently, when challenged with a two-option puzzle-box task and a paradigm allowing learning to diffuse across a population (a gold standard of cultural transmission experiments^21^, as used previously in wild great tits^22^), bumblebees were found to acquire and maintain arbitrary variants of this behaviour from trained demonstrators^23^. However, these previous investigations involved the acquisition of a behaviour that each bee could also have innovated independently. Indeed, some naive individuals were able to open the puzzle box, pull strings and roll balls without demonstrators^19,20,23^. Thus, to determine whether bumblebees could acquire a behaviour through social learning that they could not innovate independently, we developed a novel two-step puzzle box (Fig. 1a). This design was informed by a lockbox task that was developed to assess problem solving in Goffin’s cockatoos^24^. Here, cockatoos were challenged to open a box that was sealed with five inter-connected ‘locks’ that had to be opened sequentially, with no reward for opening any but the final lock. Our hypothesis was that this degree of temporal and spatial separation between performing the first step of the behaviour and the reward would make it very difficult, if not impossible, for a naive bumblebee to form a lasting association between this necessary initial action and the final reward. Even if a bee opened the two-step box independently through repeated, non-directed probing, as observed with our previous box^23^, if no association formed between the combination of the two pushing behaviours and the reward, this behaviour would be unlikely to be incorporated into an individual’s repertoire. If, however, a bee was able to learn this multi-step box-opening behaviour when exposed to a skilled demonstrator, this would suggest that bumblebees can acquire behaviours socially that lie beyond their capacity for individual innovation.

The two-step puzzle box (Fig. 1a) relied on the same principles as our previous single-step, two-option puzzle box^23^. To access a sucrose-solution reward, placed on a yellow target, a blue tab had to first be pushed out of the path of a red tab, which could then be pushed in turn to rotate a clear lid around a central axis. Once rotated far enough, the reward would be exposed beneath the red tab. A sample video of a trained demonstrator opening the two-step box is available (Supplementary Video 1). Our experiments were conducted in a specially constructed flight arena, attached to a colony’s nest box, in which all bees that were not currently undergoing training or testing were confined (Fig. 1b).

In our previous study, several bees successfully learned to open the two-option, single-step box during control population experiments, which were conducted in the absence of a trained demonstrator across 6–12 days^23^. Thus, to determine whether the two-step box could be opened by individual bees starting from scratch, we sought to conduct a similar experiment. Two colonies (C1 and C2) took part in these control population experiments for 12 days, and one colony (C3) for 24 days. In brief, on 12 or 24 consecutive days, bees were exposed to open two-step puzzle boxes for 30 min pre-training and then to closed boxes for 3 h (meaning that colonies C1 and 2 were exposed to closed boxes for 36 h total, and colony C3 for 72 h total). No trained demonstrator was added to any group. On each day, bees foraged willingly during the pre-training, but no boxes were opened in either colony during the experiment. Although some bees were observed to probe around the components of the closed boxes with their proboscises, particularly in the early population-experiment sessions, this behaviour generally decreased as the experiment progressed. A single blue tab was opened in full in colony C1, but this behaviour was neither expanded on nor repeated.

Learning to open the two-step box was not trivial for our demonstrators, with the finalized training protocol taking around two days for them to complete (compared with several hours for our previous two-option, single-step box^23^). Developing a training protocol was also challenging. Bees readily learned to push the rewarded red tab, but not the unrewarded blue tab, which they would not manipulate at all. Instead, they would repeatedly push against the blocked red tab before giving up. This necessitated the addition of a temporary yellow target and reward beneath the blue tab, which, in turn, required the addition of the extended tail section (as seen in Fig. 1a), because during later stages of training this temporary target had to be removed and its absence concealed. This had to be done gradually and in combination with an increased reward on the final target, because bees quickly lost their motivation to open any more boxes otherwise. Frequently, reluctant bees had to be coaxed back to participation by providing them with fully opened lids that they did not need to push at all. In short, bees seemed generally unwilling to perform actions that were not directly linked to a reward, or that were no longer being rewarded. Notably, when opening two-step boxes after learning, demonstrators frequently pushed against the red tab before attempting to push the blue, even though they were able to perform the complete behaviour (and subsequently did so). The combination of having to move away from a visible reward and take a non-direct route, and the lack of any reward in exchange for this behaviour, suggests that two-step box-opening would be very difficult, if not impossible, for a naive bumblebee to discover and learn for itself—in line with the results of the control population experiment.

For the dyad experiments, a pair of bees, including one trained demonstrator and one naive observer, was allowed to forage on three closed puzzle boxes (each filled with 20 μl 50% w/w sucrose solution) for 30–40 sessions, with unrewarded learning tests given to the observer in isolation after 30, 35 and 40 joint sessions. With each session lasting a maximum of 20 min, this meant that observers could be exposed to the boxes and the demonstrator for a total of 800 min, or 13.3 h (markedly less time than the bees in the control population experiments, who had access to the boxes in the absence of a demonstrator for 36 or 72 h total). If an observer passed a learning test, it immediately proceeded to 10 solo foraging sessions in the absence of the demonstrator. The 15 demonstrator and observer combinations used for the dyad experiments are listed in Table 1, and some demonstrators were used for multiple observers. Of the 15 observers, 5 passed the unrewarded learning test, with 3 of these doing so on the first attempt and the remaining 2 on the third. This relatively low number reflected the difficulty of the task, but the fact that any observers acquired two-step box-opening at all confirmed that this behaviour could be socially learned.

**Table 1:** Combinations of demonstrators and observers, with outcomes

Demonstrator ID	Demonstrator technique	Demonstrator opening incidence	Demonstrator opening index	Observer ID	Outcome	Test passed	Solo foraging phase box-opening incidence	Of which were squeezing
B-8	Squeezing	132	4.4	A-12	**✔**	1	N/A^*#*^	N/A^*#*^
B-8	Squeezing	137	3.4	A-17	**✔**	3	N/A^*#*^	N/A^*#*^
A-17^*^	Squeezing	215	7.2	A-24	**✔**	1	9	9
A-17^*^	Squeezing	187	6.2	A-6	**✔**	1	13	9
A-24^*^	Squeezing	200	5.0	A-37	***✘***	N/A	N/A	N/A
A-24^*^	Squeezing	222	5.6	A-39	***✘***	N/A	N/A	N/A
A-24^*^	Squeezing	181	4.5	C-42	**✔**	3	1	1
C-15	Staggered-pushing	158	3.9	C-26	***✘***	N/A	N/A	N/A
C-26^*^	Squeezing	72	1.8	C-19	***✘***	N/A	N/A	N/A
D-3	Staggered-pushing	227	5.7	D-11	***✘***	N/A	N/A	N/A
D-1	Squeezing	206	5.2	D-77	***✘***	N/A	N/A	N/A
D-72	Squeezing	103	3.4	D-76	***✘***	N/A	N/A	N/A
D-23	Staggered-pushing	258	6.8	D-32	***✘***	N/A	N/A	N/A
D-23	Staggered-pushing	315	7.9	D-42	***✘***	N/A	N/A	N/A
D-23	Staggered-pushing	234	7.8	D-48	***✘***	N/A	N/A	N/A

^*^Individual was originally used as an observer. In an effort to reduce training time (around two days per entirely naive bee), these bees then went through stepwise demonstrator training and were used as demonstrators for other observers when they passed the unrewarded demonstrator learning test. The letters in demonstrator and observer IDs refer to the colony from which the bee originated (A, B, C, or D), and the numbers refer to the individual tag attached to the thorax. ^#^Bee was used to trial solo foraging bout protocol, therefore results are excluded because they are incomparable. N/A, not applicable.

The post-learning solo foraging sessions were designed to further test observers’ acquisition of two-step box-opening. Each session lasted up to 10 min, but 50 μl 50% sucrose solution was placed on the yellow target in each box: as *Bombus terrestris* foragers have been found to collect 60–150 μl sucrose solution per foraging trip depending on their size, this meant that each bee could reasonably be expected to open two boxes per session^25^. Although all bees who proceeded to the solo foraging stage repeated two-step box-opening, confirming their status as learners, only two individuals (A-24 and A-6; Table 1) met the criterion to be classified as proficient learners (that is, they opened 10 or more boxes). This was the same threshold applied to learners in our previous work with the single-step two-option box^23^. However, it should be noted that learners from our present study had comparatively limited post-learning exposure to the boxes (a total of 100 min on one day) compared with those from our previous work. Proficient learners from our single-step puzzle-box experiments typically attained proficiency over several days of foraging, and had access to boxes for 180 min each day for 6–12 days^23^. Thus, these comparatively low numbers of proficient bees are perhaps unsurprising.

Two different methods of opening the two-step puzzle box were observed among the trained demonstrators during the dyad experiments, and were termed ‘staggered-pushing’ and ‘squeezing’ (Fig. 1c; Supplementary Video 2). This finding essentially transformed the experiment into a ‘two-action’-type design, reminiscent of our previous single-step, two-option puzzle-box task^23^. Of these techniques, squeezing typically resulted in the blue tab being pushed less far than staggered-pushing did, often only just enough to free the red tab, and the red tab often shifted forward as the bee squeezed between this and the outer shield. Among demonstrators, the squeezing technique was more common, being adopted as the main technique by 6 out of 9 individuals (Table 1). Thus, 10 out of 15 observers were paired with a squeezing demonstrator.

**Fig. 2:**
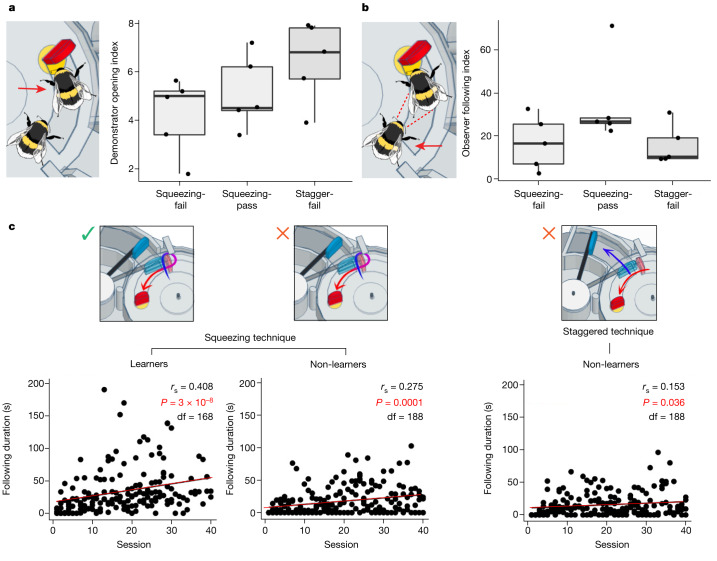
Demonstrator action patterns affect the acquisition of two-step box-opening by observers. **a**, Demonstrator opening index. The demonstrator opening index was calculated for each dyad as the total incidence of box-opening by the demonstrator/number of joint foraging sessions. **b**, Observer following index. Following behaviour was defined as the observer being present on the surface of the box, within a bee’s length of the demonstrator, while the demonstrator performed box-opening. The observer following index was calculated as the total duration of following behaviour/number of joint foraging sessions. Data in **a**,**b** were analysed using one-way ANOVA and are presented as box plots. The bounds of the box are drawn from quartile 1 to quartile 3 (showing the interquartile range), the horizontal line within shows the median value and the whiskers extend to the most extreme data point that is no more than 1.5 × the interquartile range from the edge of the box. *n* = 15 independent experiments (squeezing-pass group, *n* = 5; squeezing-fail group, *n* = 5; and staggered-pushing-fail (stagger-fail) group, *n* = 5). **c**, Duration of following behaviour over the dyad joint foraging sessions. Following behaviour significantly increased with the number of joint foraging sessions, with the sharpest increase seen in dyads that included a squeezing demonstrator and an observer that successfully acquired two-step box-opening. Data were analysed using Spearman’s rank correlation coefficient tests (two-tailed), and the figures show measures taken from each observer in each group. Data for individual observers are presented in Supplementary Fig. 1.

Although not all observers that were paired with squeezing demonstrators learned to open the two-step box (5 out of 10 succeeded), all observers paired with staggered-pushing demonstrators (*n* = 5) failed to learn two-step box-opening. This discrepancy was not due to the number of demonstrations being received by the observers: there was no difference in the number of boxes opened by squeezing demonstrators compared with staggered-pushing demonstrators when the number of joint sessions was accounted for (unpaired *t*-test, *t* = −2.015, *P* = 0.065, degrees of freedom (df) = 13, 95% confidence interval (CI) = −3.63–0.13; Table 2). This might have been because the squeezing demonstrators often performed their squeezing action several times, looping around the red tab, which lengthened the total duration of the behaviour despite the blue tab being pushed less than during staggered-pushing. Closer investigation of the dyads that involved only squeezing demonstrators revealed that demonstrators paired with observers that failed to learn tended to open fewer boxes, but this difference was not significant. There was also no difference between these dyads and those that included a staggered-pushing demonstrator (one-way ANOVA, *F* = 2.446, *P* = 0.129, df = 12; Table 2 and Fig. 2a). Together, these findings suggested that demonstrator technique might influence whether the transmission of two-step box-opening was successful. Notably, successful learners also appeared to acquire the specific technique used by their demonstrator: in all cases, this was the squeezing technique. In the solo foraging sessions recorded for successful learners, they also tended to preferentially adopt the squeezing technique (Table 1). The potential effect of certain demonstrators being used for multiple dyads is analysed and discussed in the Supplementary Results (see Supplementary Table 2 and Supplementary Fig. 4).

**Table 2:** Characteristics of dyad demonstrators and observers

Group (*n*)	Average demonsrator box-opening incidence	Significance	Average demonstrator box-opening index	Significance	Average following duration (s)	Significance	Average following index	Significance
All pass (5)	170.4		5.14		1,153		34.82	
All fail (10)	199.5	*P* = 0.414^*^ (*t* = −0.84, df = 13, 95% CI = −103.59–45.39, *d* = 0.46)	5.31	*P* = 0.867^*^ (*t* = −0.17, df = 13, 95% CI = −2.32–1.98, *d* = 0.09)	623	*P* = 0.059^*^ (*t* = −2.07, df = 13, 95% CI = −23.13–1,083.25, *d* = 1.13)	16.26	*P* = 0.055^£^ (*W* = 9)
All squeeze (10)	165.5		4.67		909		25.78	
All stagger (5)	238.4	*P* = 0.026^*^ (*t* = −2.51, df = 13, 95% CI = −135.71–10.09, *d* = 1.37)	6.42	*P* = 0.065^*^ (*t* = −2.02, df = 13, 95% CI = −3.63–0.13, *d* = 1.10)	580	*P* = 0.310^£^ (*W* = 34)	15.76	*P* = 0.281^*^ (*t* = −1.13, df = 13, 95% CI = −9.25–29.33, *d* = 0.62)
Squeezing-pass (5)	170.4		5.14		1,153		34	
Squeezing-fail (5)	160.6		4.20		665		16.75	
Stagger-fail (5)	238.4	*P* = 0.090^#^ (*F* = 2.96, df = 12, *η*^2^ = 0.33)	6.42	*P* = 0.129^#^ (*F* = 2.45, df = 12, *η*^2^ = 0.29)	580	*P* = 0.450^$^ (*χ*^2^ = 14, df = 14)	15.76	*P* = 0.114^#^ (*F* = 2.62, df = 12, *η*^2^ = 0.30)

‘Pass’ refers to dyads in which the observer passed the learning test, and ‘fail’ refers to those in which it did not. ‘Stagger’ refers to dyads including a demonstrator that preferred staggered-pushing, and ‘squeeze’ refers to dyads including a demonstrator that preferred squeezing. Dyads were further classified into ‘squeezing-pass’, ‘squeezing-fail’ and ‘stagger-fail’ groups depending on these characteristics. Data were analysed with ^*^unpaired one-sided t-tests, ^#^one-way ANOVA, ^$^Kruskal–Wallis tests or ^£^two-tailed Mann–Whitney *U* tests, depending on the number of groups and the distributions of the data, with 95% CI and effect sizes presented as appropriate. Effect sizes for parametric tests were calculated using Cohen’s *d* for *t*-tests and ETA^2^ for ANOVA. Significant comparisons are marked in bold. To account for differences in session number, the demonstrator box-opening index was calculated as the total incidence of box-opening by the demonstrator/number of joint foraging sessions. Following indexes were calculated as the total duration of following behaviour/number of joint foraging sessions. Following behaviour was defined as the observer being present on the surface of the box, within a bee’s length of the demonstrator, while the demonstrator performed box-opening (thus, following behaviour could occur only after the demonstrator began pushing the blue tab and before it accessed the reward). These figures represent the average for the group. See Table 1 for individual demonstrator box-opening data, and Supplementary Table 1 for individual observer following data.

To determine whether observer behaviour might have differed between those who passed and failed, we investigated the duration of their ‘following’ behaviour, which was a distinctive behaviour that we identified during the joint foraging sessions. Here, an observer followed closely behind the demonstrator as it walked on the surface of the box, often close enough to make contact with the demonstrator’s body with its antennae (Supplementary Video 3). In the case of squeezing demonstrators, which often made several loops around the red tab, a following observer would make these loops also. To ensure we quantified only the most relevant behaviour, we defined following behaviour as ‘instances in which an observer was present on the box surface, within a single bee’s length of the demonstrator, while it performed two-step box-opening’. Thus, following behaviour could be recorded only after the demonstrator began to push the blue tab, and before it accessed the reward. This was quantified for each joint foraging session for the dyad experiments (Supplementary Table 1). There was no significant correlation between the demonstrator opening index and the observer following index (Spearman’s rank correlation coefficient, *r*_s_ = 0.173, df = 13, *P* = 0.537; Supplementary Fig. 2), suggesting that increases in following behaviour were not due simply to there being more demonstrations of two-step box-opening available to the observer.

There was no statistically significant difference in the following index between dyads with squeezing and dyads with staggered-pushing demonstrators; between dyads in which observers passed and those in which they failed; or when both demonstrator preference and learning outcome were accounted for (Table 2). This might have been due to the limited sample size. However, the following index tended to be higher in dyads in which the observer successfully acquired two-step box-opening than in those in which the observer failed (34.82 versus 16.26, respectively; Table 2) and in dyads with squeezing demonstrators compared with staggered-pushing demonstrators (25.78 versus 15.76, respectively; Table 2). When both factors were accounted for, following behaviour was most frequent in dyads with a squeezing demonstrator and an observer that successfully acquired two-step box-opening (34.82 versus 16.75 (‘squeezing-fail’ group) versus 15.76 (‘staggered-pushing-fail’ group); Table 2).

There was, however, a strong positive correlation between the duration of following behaviour and the number of joint foraging sessions, which equated to time spent foraging alongside the demonstrator. This association was present in dyads from all three groups but was strongest in the squeezing-pass group (Spearman’s rank order correlation coefficient, *r*_s_ = 0.408, df = 168, *P* < 0.001; Fig. 2c). This suggests, in general, either that the latency between the start of the demonstration and the observer following behaviour decreased over time, or that observers continued to follow for longer once arriving. However, the observers from the squeezing-pass group tended to follow for longer than any other group, and the duration of their following increased more rapidly. This indicates that following a conspecific demonstrator as it performed two-step box-opening (and, specifically, through squeezing) was important to the acquisition of this behaviour by an observer.

## Discussion

In this article, we present evidence suggesting that *Bombus terrestris*, a social invertebrate, is capable of learning a novel behaviour from a conspecific that cannot be learned through individual trial and error. Two-step box-opening involves an initial, unrewarded step, in which bees must push a blue tab away from the path of a red tab, before pushing the red tab to receive a reward. This behaviour was so challenging that, unless an extra reward was added beneath the blue tab, demonstrators failed to learn two-step box-opening during training. This additional reward had to be removed gradually during later training stages to avoid bees refusing to open more boxes. Even so, 5 out of 15 naive observers successfully acquired the complete behaviour from the trained demonstrators. The fact that any observer bee was able to learn the complete two-step behaviour was notable precisely because they acquired the complete behaviour: these bees had never been exposed to any form of puzzle box, had not learned either of the two steps before being exposed to the other and, unlike the demonstrators, had never been rewarded for pushing the blue tab. Yet they were able to acquire the entire behaviour sequence through social learning.

By contrast, in a previous study, great tits were challenged with a two-step puzzle-box task after they had previously learned one of the two steps^26^. Although the birds could combine the two behaviours to solve the puzzle, they did not learn the complete two-step behaviour from the demonstrator: rather, they learned component behaviours and recombined them individually to form the full solution. The authors hypothesized that great tits might need both steps to be rewarded, at least initially, which mirrored the observations we made while developing our demonstrator training protocol. But bees in our study were still able to learn the complete behaviour without any reward for the first step or experience with any type of puzzle box, purely through exposure to a knowledgeable conspecific. Whether this challenge constrains multi-step social learning in other species could be worthy of further investigation.

The results of the control population experiments, in which bees were exposed to puzzle boxes for 36 h across 12 days or 72 h across 24 days, were equally telling. No bee came close to opening even a single box, and their interest in the closed boxes plummeted with time, although they continued to forage on opened boxes during pre-training. For comparison, in our previous work using the two-option puzzle box that required only a single-step action, the two 12-day control colonies generated 13 learners between them in the absence of any demonstrator^23^. This notable capacity for innovation is consistent across paradigms: in previous work on string-pulling, some bumblebees learned to pull strings without a demonstrator^19^. The failure of bees to open the two-step box independently, then, is unlikely to be due to a lack of behavioural flexibility. However, both of these previous examples involved single-step, directly rewarded tasks. If bumblebee learning relies mainly on reward- or punishment-based associations, it might simply not be possible for them to individually learn unrewarded actions, unless these are somehow linked to a rewarded action. This might be why bees succeeded in learning two-step box-opening only when exposed to a demonstrator performing the squeezing behavioural variant: this action pattern essentially combined the two steps into one, reducing the degree of temporal and spatial separation between the first step of the behaviour and the reward when compared with staggered-pushing. This may have permitted bees to form an association between the two. But the presence of the demonstrator itself was also key: observers closely following behind the demonstrator as it performed the key squeezing action essentially saw them squeezing too, accurately replicating the demonstrator’s behaviour without the need for imitative copying. Although there was no significant difference, the duration of following behaviour was also markedly increased among learners compared with non-learners, suggesting that this facilitated the transmission of two-step box-opening to an extent. Taking these lines of evidence together, bees seem highly unlikely to be capable of solving the two-step puzzle box through individual learning, even though they were capable of learning to do so socially. This is in itself impressive, but it becomes more so when one considers that it represents, to our knowledge, the first evidence of this capacity in any non-human animal. The ability to learn a behaviour socially that is too complex to be arrived at individually is still thought to represent a fundamental distinction between humans and animals, and to characterize human cumulative culture.

Cumulative culture refers to behavioural traditions that are repeatedly improved or elaborated on through sequential innovations that are socially transmitted throughout a population^1–3^. Although certain animals have been proven to be capable of sustaining such culture^16,17^, unlike in human cumulative culture, the final behaviour was not so complex that an individual could not have innovated it alone^1^. Humans are still considered the only species that is capable of socially learning behaviours that are so complex that independent re-innovation is impossible within an individual’s own lifetime^18,27^. This stipulation has resulted in a dearth of laboratory-based experimental assessments of cumulative culture in animals. It is difficult to conceive of an experimental design that might convincingly demonstrate this capacity in long-lived species, such as primates, cetaceans or corvids^28^, but these are the species that we tend to assume are most likely to be capable of this feat. Food-washing behaviours by macaques^9^, pandanus-leaf tool designs by New Caledonian crows^12^ and the songs of humpback whales^29^ have all been proposed as potential examples of cumulative culture, but none have been confirmed through laboratory-based experiments. In the case of cetaceans, there are additional physical constraints: it is hard to see how one would even begin to approach such an experiment in a wild humpback whale. This does not mean that these animals are incapable of cumulative culture, or even that these examples do not represent it: it simply means that we cannot know for sure whether they do. Even with our present study, we cannot rule out the possibility that one bee in a million might manage to solve the two-step box within its lifetime, although this seems unlikely. Adult bumblebees do not dedicate their entire lifespan to foraging: younger adults typically remain in the nest, performing tasks such as nursing or building, with older adults becoming foragers and tending to remain so until their death^30,31^. Foraging lifespan does vary between individuals, with one previous study finding an average of 8.33 days (range, 1–22 days, *n* = 49 bees)^31^. This would fall well within the duration of our control experiments.

In this study, we did not aim to examine the maintenance of two-step box-opening as a behavioural tradition by a group of bumblebees over time, as we did in our previous work with the two-option box^23^. However, the evidence gathered by the present study and our previous work suggests that this would at least be cognitively plausible. This is especially notable because *Bombus terrestris* has not been confirmed to exhibit any form of culture in the wild, cumulative or otherwise, although nectar-robbing and its laterality could represent strong candidates for non-cumulative culture^32,33^. In fact, on the surface, this species seems unlikely to show cumulative culture at all in the wild. Bumblebee colonies in temperate regions do not typically persist beyond a single biological generation^30^. They are survived by naive queens that might have little opportunity to learn from experienced individuals, which would effectively reset any annual accumulation of complexity or efficiency. Still, in our study, bumblebees were able to learn a behaviour from a demonstrator that was complex enough that they failed to re-innovate it independently. Perhaps, then, the apparent lack of cumulative culture among wild bumblebees is as simple as a lack of opportunity and need^34^. Wild bees are perhaps unlikely to stumble across natural analogues of multi-step puzzle boxes that they must open to feed. But why they are capable of such a feat, if it is not something necessary in their natural lives, remains an unanswered question.

Social insects have some of the richest, most intricate behavioural repertoires in the entire animal kingdom. Their nest architectures are orders of magnitude larger than any individual, and are built in common. Leafcutter ants farm fungus on the leaves that they collect^35^, and honeybees communicate the distance and direction of resources through their dance language^36^. This behaviour was all once thought to be purely instinctive. However, we are increasingly beginning to appreciate the role of social learning in such behaviour: at least some components of the honeybee dance language appear to be shaped by social influences^37^. Some social insect species form colonies that last for years or even decades: these include honeybees^38^, tropical bumblebees^39–41^ and stingless bees^42,43^. If the learning abilities of these species resemble those of *Bombus terrestris*, these might be the best candidates in which to observe the natural occurrence of culture—even cumulative culture—and they represent exciting models through which further questions can be pursued.

## Methods

### Animal model

Bumblebees (*Bombus terrestris audax*) were obtained from Agralan. Whole colonies were transferred to 30.0 × 14.0 × 16.0-cm bipartite wooden nest boxes after delivery, and were housed in these for the duration of the experiment. Colonies were maintained at room temperature, and experiments were performed under standardized artificial lights (12:12, high-frequency fluorescent lighting; TMS 24F lamps with HF-B 236 TLD (4.3 kHz) ballasts (Koninklijke Phillips), fitted with Activa daylight fluorescent tubes (OSRAM Licht)). Bees foraged ad libitum on 20% w/w sucrose solution, which was provided by means of mass feeders overnight in the flight arena, with pollen provided directly to the nest box every two days. All individuals used for our experiments were female workers, and while the specific age of each individual was not determined they were of an age typical of foragers for this species. None of the colonies had been used for any previous experiments. All experiments were conducted in accordance with the ASAB/ABS guidelines for the use of animals in research. There were no licence or permit requirements for the experiments presented in this paper.

### Experimental set-up and puzzle-box design

#### Puzzle box

The two-step puzzle box was a modified version of the two-option puzzle box used in our previous study^23^. This incorporated a transparent lid that rotated anti-clockwise around a central axis when a red tab was pushed, exposing a 50% w/w sucrose-solution reward on a yellow target; however, an additional blue tab initially blocked the path of the red tab (Fig. 1a). Two stoppers prevented either tab from being moved in the incorrect direction, and a plastic shield around the box prevented bees reaching with their proboscis from the side of the box to obtain the reward without manipulating the tabs.

#### Flight arena

All experiments were performed in a flight arena (66.0 × 60.0 × 30.0 cm), which was connected to the hive boxes with transparent acrylic tunnels (26.0 × 3.5 × 3.5 cm). Plastic strips used as sliding doors along this tunnel controlled access to the flight arena. Flaps cut into the arena sides allowed the removal and replacement of puzzle boxes with minimal disturbance to the bees, and brush strips lining the sides of the flight arena prevented bees from escaping during this process. The interior walls were lined with laminated paper showing a random distribution of pink dots. The top of the flight arena was a sheet of transparent UV-transmitting perspex acrylic, and cameras (iPhone 6S; Apple) were placed on top of the arena to record all experiments from above.

#### Individual tagging

Bees were marked with numbered Opalith tags (Opalithplättchen; Bienen-Voigt & Warnholz) for individual identification^23^. In brief, small groups of bees were allowed into the flight arena to forage on lidless puzzle boxes, with yellow targets (carrying 10 μl 50% w/w sucrose-solution rewards) fully exposed and accessible. Bees that foraged from multiple boxes (that is, were motivated foragers) were captured and tagged before being returned to the hive box. Bees were never used for experiments on the same day that they were tagged to prevent any confounding stress-associated effects, and to allow acclimatization to the tag.

### Demonstrator training protocol

#### Manual training of two-step box-opening

Potential demonstrators were identified during group foraging on lidless boxes, as described above. When a tagged bee was observed repeatedly and reliably coming back and forth between the nest box and the flight arena to forage, it was selected for further training, and all other bees were restricted to the nest box. Full details of the stepwise protocol can be found in Supplementary Fig. 3. Notably, to train bees to push the unrewarded blue tab as the first step of the behaviour, it was necessary to introduce a temporary additional reward beneath this tab, which was then removed during later training stages. Successful acquisition of two-step box-opening was confirmed with an unrewarded learning test.

#### Demonstrator unrewarded learning test

Once a bee reliably opened two-step puzzle boxes in exchange for no reward beneath the blue tab and for 20 μl 50% w/w sucrose solution beneath the red tab, it proceeded to the unrewarded learning test. The full unrewarded learning test protocol can be found in the Supplementary Information.

A total of 13 bees from four colonies (colony IDs A–D) passed the demonstrator learning test. Three of these (A-8, A-6 and A-27) were used to pilot these experiments, and one (B-25) died before it could be used as a demonstrator. Thus, nine bees in total were used as demonstrators for the dyad experiments (Table 1). The average training period for a wholly naive bee was two days and, in an attempt to reduce this time, three trained demonstrators (A-17, A-24 and C-26) were originally used as observers in the dyad experiments before being put through the stepwise training protocol and passing the unrewarded learning test.

### Dyad experiments

#### Observer selection

In total, 15 observers from three colonies took part in these experiments. Each observer was paired with a trained demonstrator, with some demonstrators being used for multiple observers in succession (Table 1). Observers were selected in the same manner as demonstrators, during group foraging on lidless boxes, with only the most motivated and reliable foragers chosen. Thus, although observers were familiar with the yellow target indicating the presence of a reward, they had no experience with closed boxes, or with the movement of the tabs. They also never had any experience of being rewarded for pushing the blue tab, as demonstrators did during early training phases.

#### Joint foraging phase

Before each joint foraging session, the demonstrator and the observer were held in the tunnel for 3 min, starting from when both were present. After this, they were released into the flight arena and presented with three closed puzzle boxes, each filled with 20 μl 50% w/w sucrose solution. As these were opened, they were removed from the arena, cleaned with 70% ethanol to remove olfactory cues, refilled and replaced. Once the demonstrator returned to the tunnel (or after 20 min elapsed, at which point the demonstrator was manually returned to the nest box) the experimenter removed one box, opened it and placed it back in the arena so that the observer could access the reward. This was again intended to preserve foraging motivation. Occasionally, demonstrators lost motivation to forage during this phase: to counter this, if they ever failed to open more than two boxes in a single session, they were given individual foraging sessions until they opened boxes consistently again.

#### Observer unrewarded learning test

After 30 joint foraging sessions, the observer proceeded to an unrewarded learning test in the absence of the demonstrator. This was identical to the test used for demonstrators, but if the box was opened in the 15-min time limit, the observer was given a yellow acrylic chip loaded with 10 μl 50% w/w sucrose solution followed by a closed box with 50 μl 50% w/w sucrose solution on the target. The observer was then allowed to forage ad libitum on closed puzzle boxes (containing the usual 20 μl 50% w/w sucrose solution), until it either returned to the nest box or stopped opening boxes for 3 min. Observers that passed proceeded to the solo foraging phase, whereas those that failed returned to joint foraging sessions with the demonstrator. Additional learning tests were given after every five ‘remedial’ joint foraging sessions, but if the observer failed three times it was considered to have failed to acquire two-step box-opening. At this point, joint foraging was ceased.

#### Solo foraging phase

Observers that passed the learning test proceeded to the solo phase. Here, they were challenged with ten foraging sessions in the absence of the demonstrator. Each session was preceded by a 3-min waiting period in the tunnel and then lasted up to 10 min, after which the bee was manually returned to the nest box if it had not already returned of its own accord. Three puzzle boxes, each containing 50 μl of 50% w/w sucrose solution, were placed in the arena, and if none were opened during a session, the bee was given a yellow acrylic chip with 10 μl of 50% w/w sucrose solution before being returned to the nest box. This was done in an effort to maintain their foraging motivation for further sessions.

### Control population experiments

A total of three colonies (IDs C1, C2 and C3) were used for these experiments, which followed a similar protocol to that used with the control population described in our previous work^23^. These experiments aimed to determine whether bees could spontaneously learn to open the two-step box during an extended period of exposure. Several bees managed to open a single-step puzzle box without a demonstrator in our previous experiments^23^.

In brief, each day at around 9.30 a.m., the mass feeders were removed from the flight arena and the bees were returned to the nest box. If more than two honeypots were full, the sucrose solution was removed using a handheld pipette, with care being taken to avoid damaging any part of the hive structure. This ensured a strong motivation to forage. After around 30 min, bees were allowed unrestricted access to the flight arena, in which they received 30 min of group pre-training with eight lidless boxes, with the yellow targets (bearing 10 μl w/w 50% sucrose rewards) fully exposed. This daily pre-training ensured that the bees maintained a strong association between the colour yellow and the reward, and encouraged as many bees into the flight arena as possible before the experiment began. After the pre-training, the boxes were removed, wiped with 70% ethanol, refilled with 20 μl w/w 50% sucrose, closed and replaced to begin the population experiment. Each experimental session lasted for 3 h. Colonies C1 and C2 underwent the experiment for 12 consecutive days (36 h total exposure to the closed boxes), and colony C3 underwent the experiment for 24 consecutive days (72 h total exposure to the closed boxes).

### Video analysis

All videos were analysed using BORIS v.7.10.2, which permitted the coding of point events, the extraction of event durations and the assigning of each event to a bee ID, box ID, tab colour and opening method as appropriate^44^.

#### Dyad experiments

For both the joint sessions and the post-learning solo foraging sessions, point events were coded when boxes were opened (‘demonstrator opening incidence’ in the joint sessions; ‘learner opening incidence’ in the solo sessions). The start and end of observer ‘following behaviour’ were also coded as point events in the joint sessions. Following behaviour was defined as the observer being present on the surface of the puzzle box, within a bee’s length of the demonstrator, while it performed box-opening (so following behaviour could occur only after the demonstrator began pushing the blue tab and before it accessed the reward). The total duration of following behaviour in each joint session was thus extracted.

As the dyads underwent different numbers of joint sessions, depending on whether the observer acquired two-step box-opening or not, demonstrator opening incidence and observer following duration were indexed to permit statistical comparisons. The demonstrator opening index for each dyad was calculated as the total incidence of box-opening/the number of joint sessions, and the following index was calculated as the total duration of following/the number of joint sessions.

#### Box-opening method

As mentioned above, two different methods to open the two-step puzzle box were identified during these experiments: squeezing and staggered-pushing (Fig. 1c). Thus, all incidences of box-opening in both the dyad and the control population experiments were labelled with the box-opening technique that was used. Because in some cases, bees would leave the box surface during the opening or move between the blue and red tabs several times, even while performing the key squeezing movement between the tabs (sometimes repeatedly), any openings in which this squeezing action was incorporated were labelled ‘squeezing’.

### Statistical analysis

Data were analysed using R (v.4.0.4)^45^. Comparisons between two groups were made with unpaired one-sided *t*-tests or Mann–Whitney *U* tests, depending on the normality of the data (assessed using Shapiro–Wilk tests), and homogeneity of variances (assessed using F tests). Comparisons between more than two groups were made with one-way ANOVA or Kruskal–Wallis tests, as appropriate. Correlation analysis was performed using Spearman’s rank order correlation coefficient tests. Sample size selection was based on those used in other comparable studies, and as observers were assigned into groups based on whether they passed or failed an unrewarded learning test and the predominant behavioural variant used by their demonstrator, randomization was not applicable. This was an observational study of animal behaviour and so did not require blinding. *P* < 0.05 was considered to indicate a statistically significant difference.

### Reporting summary

Further information on research design is available in the Nature Portfolio Reporting Summary linked to this article.

## Online content

Any methods, additional references, Nature Portfolio reporting summaries, source data, extended data, supplementary information, acknowledgements, peer review information; details of author contributions and competing interests; and statements of data and code availability are available at 10.1038/s41586-024-07126-4.

### Supplementary information

Supplementary Information This file contains Supplementary methods; Supplementary results, Supplementary figures and Supplementary tables.

Reporting Summary

Supplementary Video 1 Sample video of trained demonstrator B-8 opening the two-step box.

Supplementary Video 2 Examples of ‘squeezing’ and ‘staggered-pushing’box-opening variants. Examples of both continuous and non-continuous ‘staggered-pushing’are shown.

Supplementary Video 3 Sample video showing 'following' behaviour. Trained demonstrator B-8 opens the box, while observer A-12 ‘follows’ its behaviour. ‘Following’ behaviour is defined as the observer walking behind the demonstrator on the surface of the box, often close enough to contact its body with its antennae.

## Data Availability

Data underlying the figures presented in the manuscript are available at 10.6084/m9.figshare.25012286.
