# Supplementary material for: Bumblebees socially learn behaviour too complex to innovate alone
Source: Nature. 2024 Mar 6;627(8004):572–8. doi: 10.1038/s41586-024-07126-4 (PMC10954542; doi:10.1038/s41586-024-07126-4)
Supplement: Supplementary file 1 — This file contains Supplementary methods; Supplementary results, Supplementary figures and Supplementary tables. [file 41586_2024_7126_MOESM1_ESM.pdf]

nature portfolio

https://doi.org/10.1038/s41586-024-07126-4

Supplementary information

Bumblebees socially learn behaviour too complex to innovate alone

In the format provided by the authors and unedited

Supplementary information

Supplementary methods

Puzzle box design elements. Box bases were 3D-printed to ensure consistency, and the surface was covered in laminated grey RGB-neutral paper (hex #555555), which was lightly sanded to provide grip. A Petri dish lid served as the rotating lid of the box, with one cut-out track available for bees to use. A second cut-out track was made in the lid for balance but was covered with a thin sheet of clear plastic. White acrylic “washers” and nails formed the two rotating mechanisms. The red and blue tabs were cut from 2 mm-thick polyethylene craft foam sheets, and while the red tab was affixed to the front of the cut-out track on the lid, the blue tab was attached to a thin strip of plastic. The other end of this plastic “arm” was incorporated into the rotating mechanism on the column. An opaque cover, made from laminated grey paper, was cut so that it would follow the curve of the tail section and was affixed to the bottom of the blue tab, as depicted. The yellow target indicated the location of the reward (50% w/w sucrose solution) and was always visible, but inaccessible without opening the box. The outer “shield” surrounding the box prevented bees obtaining rewards by squeezing under the lid from the sides, and two stoppers prevented the movement of either tab in inappropriate directions.

Stepwise demonstrator training protocol. The stepwise demonstrator training protocol is presented in Supplementary Figure 3. During training, all boxes were washed with 70% ethanol after depletion and before replacement, to ensure the removal of any olfactory cues. The first stage of training (Supplementary Fig. 3A) involved training the demonstrator to push the blue tab out of the way of the red tab: during this stage, a temporary yellow target and sucrose solution reward was added beneath the blue tab.

Once a demonstrator could reliably access the initial reward beneath the blue tab (from Supplementary Fig. 3A, position iii) they proceeded to the second stage (Supplementary Fig. 3B). From this point, only one box was presented at a time to discourage bees from pushing multiple blue tabs without trying to push any red tabs. The initial reward was reduced from 20 to 10 μl 50% w/w sucrose solution, and an additional 10 μl 50% w/w sucrose solution reward was added to the final yellow target. Once the bee had obtained the reward beneath the blue tab, pushing it out of the way of the red tab in the process (Supplementary Fig. 3B inset), the experimenter used tweezers to manipulate the position of the red tab with respect to the second reward. As with the first reward, this was made progressively harder to obtain (Supplementary

Fig. 3B i-iii), until the red tab was set far enough back that it was blocked by the blue tab and no longer required experimenter interference (Supplementary Fig. 3B iv).

At this point, the temporary reward beneath the blue tab was removed, but this had to be done slowly and progressively. When bees found no reward beneath the blue tab, they would soon refuse to open any more boxes, necessitating provision with boxes with the temporary target and reward reinstated (and easily accessible). Initially, one reward was removed per three boxes presented, then two per four boxes, then two per three boxes, until finally the temporary reward was removed entirely. The sucrose reward beneath the red tab also had to be tripled to 30 µl during this stage, or bees became reluctant to open more boxes. The final stage of training involved the reduction of this reward back to 20 µl, to ensure that demonstrators would not be satiated too quickly in subsequent experiments, and maximise the number of their demonstrations. If a bee stopped opening boxes at any point, it was presented with "easier" configurations from earlier training stages until it resumed its box-opening activity. Once a bee opened two boxes in the final configuration in exchange for 20 µl 50% sucrose solution, they proceeded to the unrewarded learning test.

Unrewarded learning test. Here, the bee was presented with a single, closed box with distilled water in place of the sucrose reward, to avoid any confounding olfactory cues. If the box remained unopened after 10 min had elapsed, a yellow acrylic chip bearing 10 µl 50% w/w sucrose solution was placed in the arena to retain foraging motivation. This chip was removed when depleted, and an additional 5 min were given to open the box. The increased duration of the test compared with that used for the two-option puzzle box task in our previous work[23] was intended to reflect the increased difficulty of the two-step box. Bees that failed to open the box within the time limit returned to training until they met the unrewarded test criterion again. Bees that opened the box within the time limit 'passed' the test, and to prevent refusal to open further boxes in response to the distilled water, were immediately provided with a yellow acrylic chip carrying 10 µl 50% w/w sucrose solution. The experimenter replaced the opened box with a closed one containing 20 µl 50% w/w sucrose solution on the target, and the bee was then allowed to solve rewarded puzzle boxes *ad libitum* until it attempted to leave the flight arena.

Supplementary results

Demonstrator experience may have influenced observer following behaviour. Due to the long, difficult demonstrator training protocol, some demonstrators were used with multiple observers in sequence, if they were still active after completing a dyad. A total of 10/15 observers were paired with a demonstrator used for more than one observer, and 5/15 observers were paired with a demonstrator used solely with them. There was a mild significant difference in demonstrator opening index between these two groups, with slightly more boxes being opened in dyads where the demonstrator was used repeatedly compared with those where it was used only once (unpaired one-sided t-test, t=2.219, df=13, p=0.045; Supplementary Fig. 4A and Supplementary Table 2). However, when we compared dyads where the demonstrator was being used for the first time (9/15 cases) with dyads where the demonstrator had been used previously (6/15 cases), this difference was attenuated (unpaired one-sided t-test, t=-1.1853, df=13, p=0.2571; Supplementary Fig. 4A and Supplementary Table 2). This suggested that the demonstrators were not becoming more proficient at box-opening over time: rather, demonstrators that were used again after completing a dyad were a little more proficient than those who were not. Considering demonstrators were only used a second time if they were still active and motivated to open boxes, this is perhaps unsurprising.

Interestingly, in terms of observer following behaviour, there was a strong significant difference in following between observers paired with demonstrators who were used multiple times, and those paired with demonstrators used just once (Mann Whitney U test, W=1, p=0.001; Supplementary Fig. 4B and Supplementary Table 2). As with the demonstrator opening index, this difference was attenuated when comparing between first dyads and subsequent dyads (Mann Whitney U test, W=21, p=0.529; Supplementary Fig. 4B and Supplementary Table 2), suggesting that increased observer following was inherently linked to these specific demonstrators: it did not increase with demonstrator experience. But following is by definition an *observer* behaviour, not a demonstrator behaviour. One explanation may be that these demonstrators were more tolerant of the observer following them, continuing to demonstrate for longer after the observer arrived to follow.

This finding is particularly notable when we consider that all observers who passed the learning test were paired with a demonstrator who was used repeatedly (which meant they spent more time following them), *and* who preferred to use the "squeezing" technique (n=5). However, not all observers paired with a demonstrator used repeatedly passed (n=5), even when this demonstrator preferred "squeezing" (n=2; with n=3 observers paired with a repeatedly-used demonstrator who preferred "staggered-pushing"). Thus, while we cannot say

for certain that these demonstrator characteristics *will* elicit learning in an observer, they may be necessary for learning to arise.

Supplementary figures

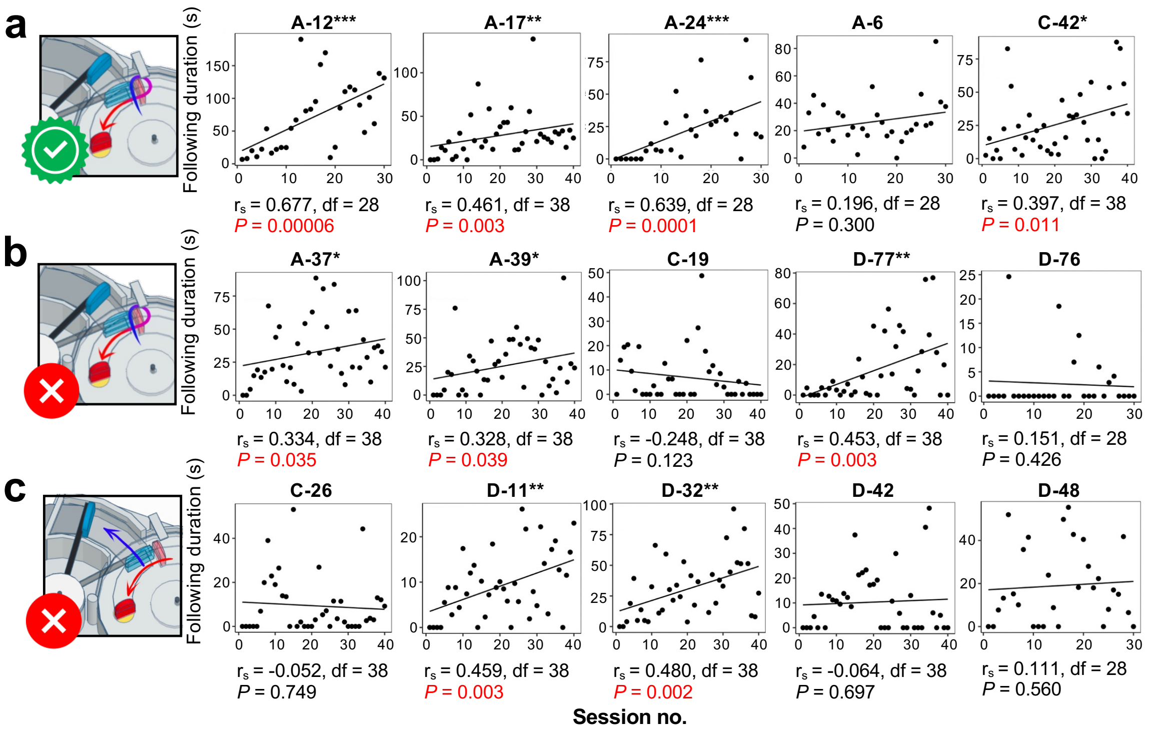

Supplementary Figure 1. Following duration over the dyad joint foraging sessions for individual dyads. ***(A)*** *Dyads where the observer acquired two-step box-opening and the demonstrator preferred the squeezing technique.* ***(B)*** *Dyads where the observer failed to acquire two-step box-opening and the demonstrator preferred the squeezing technique.* ***(C)*** *Dyads where the observer failed to acquire two-step box-opening and the demonstrator preferred the squeezing technique. Data were analysed using Spearman's rank correlation coefficient tests (two-tailed), and significant results are highlighted in red. Graph titles refer to the observer ID.*

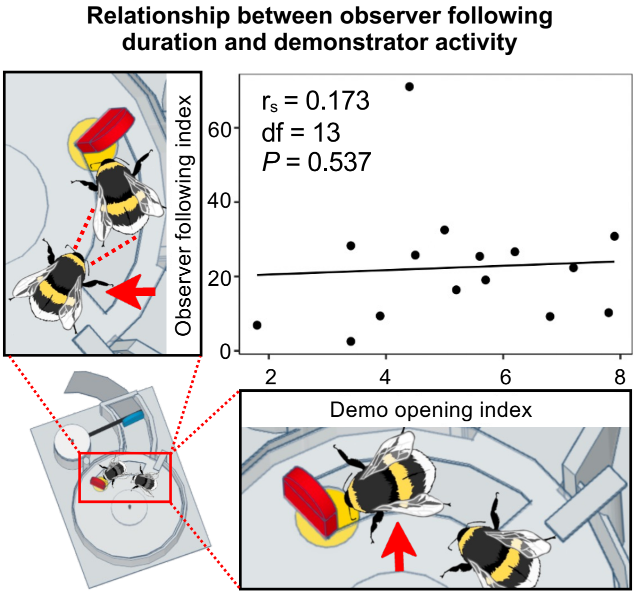

***Supplementary Figure 2. There was no significant correlation between demonstrator opening index and observer following index in the dyads**. This suggested that increases in following behaviour were not simply due to there being more demonstrations of two-step box-opening available to the observer. To account for differences in session number, demonstrator box-opening indexes were calculated as the total incidence / number of sessions. Following indexes were calculated as the total duration of following behaviour / number of sessions. Data were analysed using a Spearman's rank correlation coefficient test (n=15).*

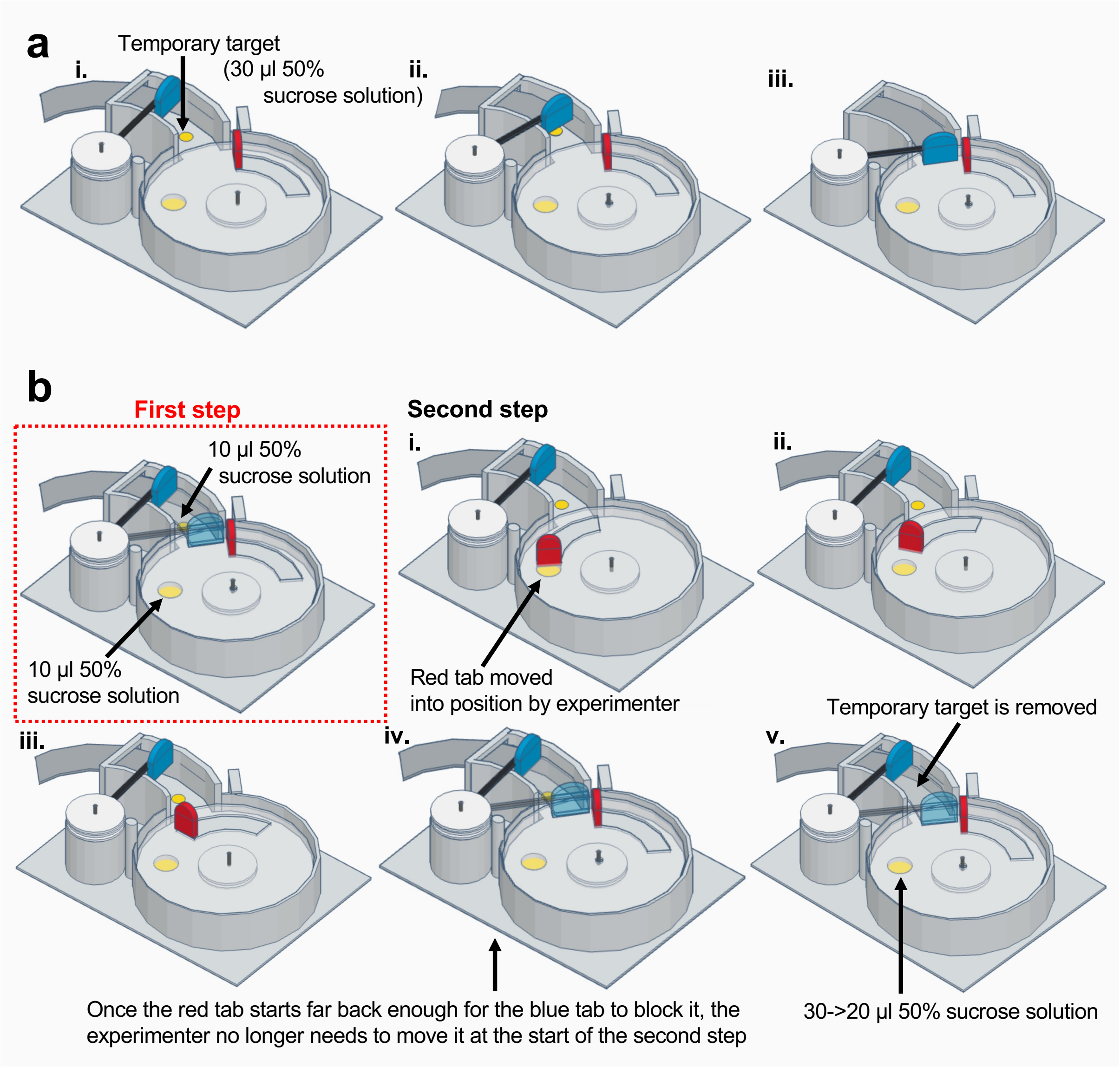

Supplementary Figure 3. Stepwise demonstrator training. (A) *Training a demonstrator to push the blue tab. A temporary yellow target bearing 30 µl 50% sucrose solution was added to the tail section of the box; the blue tab was initially positioned so that this was fully exposed (i). Once the bee learned the location of the reward, the blue tab was moved further over the target. Initially, the reward could still be obtained by reaching under the tab, but the tab was often pushed forward as the bee attempted this (ii). This continued until the reward was inaccessible without pushing, and the blue tab blocked the red tab (iii).* ***(B)*** *Training a demonstrator to push the red tab. Yellow targets now bore 10 µl 50% sucrose solution. When the bee reliably pushed the blue tab from the fully-closed configuration (A iii), phase 2 of training began. Once bees pushed the blue tab and obtained the reward, , the experimenter used tweezers to move the red tab forwards and expose the second yellow target with reward (second step; i). As above, the location of the*

red tab in the second step was progressively shifted so that the second yellow target was increasingly inaccessible (ii-iii). Once the bee was able to push the red tab from a position far enough back that the blue tab blocked it when closed, the experimenter no longer intervened before the step 2 (iv). The final step was the removal of the temporary yellow tab beneath the blue door and a temporary increase of the reward to 30 µl 50% sucrose solution, decreased to 20 µl once the bee reliably opened the box by pushing both tabs with no reward beneath the blue tab. Once it reliably did this for 20 µl, it progressed to the learning test.

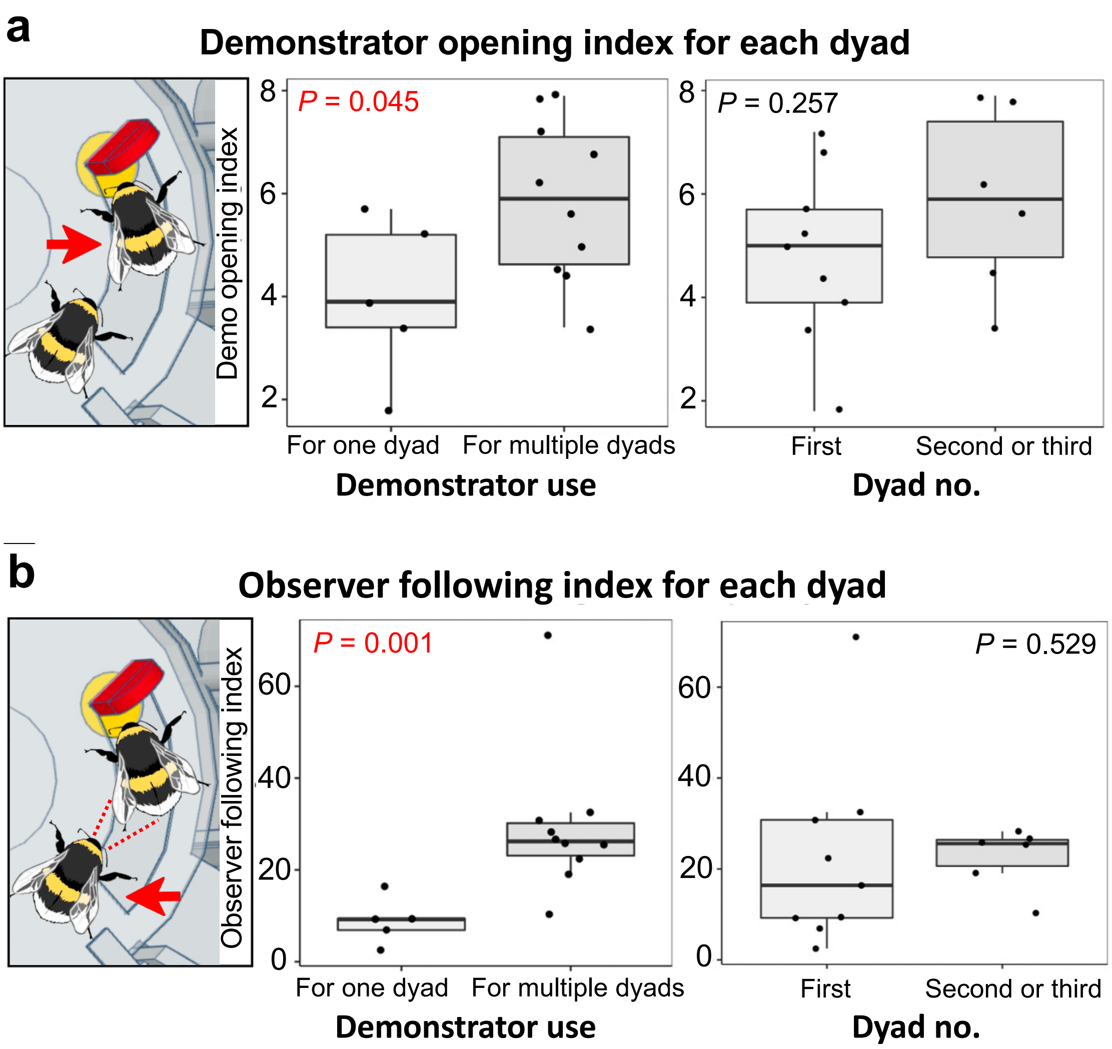

Supplementary Figure 4. Demonstrator experience may have influenced observer following behaviour. *The left-hand graphs in each panel show the dyads grouped according to whether the demonstrator was used for one or multiple dyads. The right-hand graphs show the dyads grouped according to whether this was the demonstrator's first dyad, or whether it had been used in a previous dyad.* ***(A) Demonstrator opening index.*** *The demonstrator opening index was calculated for each dyad as the total incidence of box-*

opening by the demonstrator / number of joint foraging sessions. Data were analysed using an unpaired one-sided t-test. ***(B) Observer following index.*** *Following behaviour was defined as the observer being present on the surface of the box, within a bees' length of the demonstrator, while the demonstrator performed box opening. The observer following index was calculated as the total duration of following behaviour / number of joint foraging sessions. Data were analysed using a Mann-Whitney U test, due to an abnormal data distribution. Data in (A) and (B) were taken from n=15 independent experiments (one dyad, n=5, multiple dyads, n=10; first dyad, n=9, second/third dyad, n=6) and are presented as box plots. The bounds of the box are drawn from* Q_1 *to* Q_3 *(showing the interquartile range), the horizontal line within shows the median value, and the whiskers extend to the most extreme data point that is no more than 1.5 x the interquartile range from the edge of the box.*

Supplementary tables

Supplementary Table 1. Duration of observer following behaviour in the dyad experiments.

	Following time (s)														
	Learners					Non-learners (squeezing)					Non-learners (staggered pushing)				
**Demo ID**	**B-8**	**B-8**	**A-17**	**A-17**	**A-24**	**A-24**	**A-24**	**C-26**	**D-1**	**D-72**	**C-15**	**D-3**	**D-23**	**D-23**	**D-23**
**Obs. ID**	**A-12**	**A-17**	**A-24**	**A-6**	**C-42**	**A-37**	**A-39**	**C-19**	**D-77**	**D-76[#]**	**C-26**	**D-11**	**D-32**	**D-42**	**D-48[#]**
**Session no. 1**	6.7	0.0	0.0	8.0	2.5	0.0	0.0	0.0	0.0	0.0	0.0	0.0	0.0	0.0	0.0
**2**	8.0	0.0	0.0	33.0	15.2	0.0	0.0	14.0	4.7	0.0	0.0	0.0	0.0	0.0	0.0
**3**	20.0	0.9	0.0	45.8	0.0	4.5	0.0	19.4	0.0	0.0	0.0	0.0	4.0	0.0	7.7
**4**	10.8	14.0	0.0	17.7	6.3	14.8	4.4	20.4	0.0	0.0	0.0	0.0	18.9	4.5	13.2
**5**	22.3	10.8	0.0	38.8	0.0	19.2	19.9	9.5	0.0	24.6	0.0	5.5	39.3	0.0	52.0
**6**	53.5	20.6	0.0	20.5	22.4	13.5	18.0	1.5	4.8	0.0	6.9	8.8	5.0	13.5	15.2
**7**	16.3	0.4	6.2	12.3	82.7	17.5	76.0	19.6	0.0	0.0	19.9	2.8	13.7	0.0	10.1
**8**	22.0	3.8	11.7	33.0	54.5	67.5	0.0	0.0	4.4	0.0	39.0	8.8	5.0	12.7	35.8
**9**	24.9	30.5	7.0	30.8	7.0	19.7	4.5	0.0	9.0	0.0	22.9	4.4	4.0	11.2	41.5
**10**	24.8	12.5	6.0	16.8	24.5	43.8	0.0	0.0	3.3	0.0	19.0	17.4	32.5	10.8	0.0
**11**	54.2	0.0	27.8	22.4	2.5	51.8	34.1	3.5	0.0	0.0	26.5	7.4	66.3	9.5	0.0
**12**	67.0	51.7	7.0	2.5	15.8	22.5	29.8	0.0	2.4	0.0	13.9	12.0	11.0	13.8	0.0
**13**	190.3	22.0	52.2	21.4	32.8	10.3	20.9	0.0	7.2	0.0	13.5	13.7	13.5	12.2	23.9
**14**	80.8	87.1	1.5	16.4	14.0	20.9	0.0	12.7	0.0	0.0	0.0	0.0	59.2	8.5	8.8
**15**	83.4	14.7	33.2	52.1	21.0	8.3	13.5	6.3	2.1	18.5	53.1	10.2	30.2	37.4	0.0
**16**	95.2	21.5	22.5	31.5	8.8	39.0	13.0	6.3	23.6	0.0	0.0	2.3	21.5	21.3	49.8
**17**	151.8	58.6	17.8	26.0	25.0	3.0	47.2	0.0	11.9	0.0	2.0	7.2	33.8	22.2	55.4
**18**	169.8	12.0	76.4	16.1	6.0	54.2	26.8	0.0	1.1	7.0	0.0	18.4	24.4	23.3	42.8
**19**	9.5	29.5	36.7	19.2	3.5	32.1	29.5	0.0	0.0	12.5	0.0	8.8	52.9	17.2	18.2
**20**	25.2	38.0	26.5	0.0	11.2	62.9	15.5	22.1	45.2	0.0	0.0	10.1	3.8	17.5	40.5
**21**	85.2	42.8	29.1	12.0	3.1	88.6	35.7	0.0	0.0	0.0	3.0	8.5	41.5	19.2	28.0
**22**	110.3	43.0	32.6	18.5	44.0	31.8	48.5	0.0	12.6	0.0	26.9	5.8	17.5	0.0	18.0
**23**	117.5	59.8	30.1	20.4	15.9	80.5	48.7	27.3	42.0	6.0	5.3	0.0	36.6	0.0	22.3
**24**	112.9	11.2	35.7	24.7	32.2	51.7	59.3	48.7	56.4	0.0	7.1	9.7	n/a[$]	0.0	0.0
**25**	89.8	11.7	19.2	46.6	31.2	22.0	44.3	17.7	13.8	2.8	0.0	5.7	n/a[$]	10.7	8.0
**26**	48.0	18.8	0.0	23.8	33.0	83.8	6.8	9.3	31.8	4.1	11.5	26.1	11.5	29.9	17.0
**27**	101.5	32.3	91.9	25.3	48.3	34.8	49.3	11.8	45.6	0.0	3.5	21.7	42.5	5.8	15.0
**28**	61.3	55.5	62.8	85.1	27.3	16.5	41.4	4.0	41.6	0.0	11.4	1.9	19.1	0.0	41.8
**29**	138.2	138.8	19.3	40.9	13.9	8.0	40.2	8.5	4.3	0.0	2.0	7.9	37.9	0.0	6.5
**30**	131.2	21.0	17.0	37.6	57.5	63.5	48.1	3.0	3.8	0.0	0.0	4.8	33.0	13.0	0.0
**31**	n/a	29.5	n/a	n/a	0.0	21.3	0.0	0.0	0.0	n/a	0.0	22.2	72.5	0.0	n/a
**32**	n/a	26.0	n/a	n/a	14.2	64.0	29.2	0.0	15.8	n/a	0.0	14.1	44.5	0.0	n/a
**33**	n/a	23.8	n/a	n/a	0.0	20.8	8.0	0.0	28.5	n/a	0.0	3.0	96.0	0.0	n/a
**34**	n/a	20.0	n/a	n/a	5.5	42.0	14.0	5.3	75.8	n/a	44.3	17.2	52.2	40.5	n/a
**35**	n/a	32.0	n/a	n/a	53.5	28.6	2.0	0.0	39.5	n/a	2.6	19.1	51.3	48.2	n/a
**36**	n/a	29.7	n/a	n/a	33.8	10.0	23.5	4.5	76.9	n/a	3.7	12.7	79.9	6.0	n/a
**37**	n/a	33.2	n/a	n/a	87.7	36.0	102.4	0.0	27.9	n/a	3.0	0.0	51.4	0.0	n/a
**38**	n/a	14.3	n/a	n/a	82.9	37.3	11.3	0.0	0.0	n/a	13.0	11.5	8.9	5.0	n/a
**39**	n/a	34.0	n/a	n/a	56.3	32.9	27.4	0.0	19.8	n/a	12.1	16.6	8.0	0.0	n/a
**40**	n/a	25.0	n/a	n/a	34.0	21.2	23.7	0.0	0.0	n/a	9.2	23.0	27.3	0.0	n/a
Total	***2132.4***	***1131.0***	***670.2***	***799.2***	***1030.0***	***1300.8***	***1016.9***	***275.4***	***655.8***	***75.5***	***375.3***	***369.3***	***1170.6***	***413.9***	***571.5***
Group av.					***1152.6***					***664.88***					***580.12***
Following index	***71.08***	***28.28***	***22.34***	***26.64***	***25.75***	***32.52***	***25.42***	***6.89***	***16.39***	***2.52***	***9.38***	***9.23***	***30.81***	***10.35***	***19.05***
Group av.					***34.82***					***16.75***					***15.76***

[#]Did not complete all joint sessions due to either demonstrator or observer death. [$]Video file corrupted and unusable. To account for differences in session number, following indexes were calculated as the total duration of following behaviour / number of sessions.

Supplementary Table 2. Dyad demonstrator and observer characteristics.

Group (n)	Av. demo box-opening incidence	Sig.	Av. demo box-opening index	Sig.	Av. following duration (s)	Sig.	Av. following index	Sig.
**Repeat (10)**	208.1		5.88		1024		29.22	
**Non-repeat (5)**	153.2	$p=0.110^{*}$ (t=1.71, df=13, 95% CI= -14.17 - 123.97, d=0.94)	4.00	**$p=0.045^{*}$** (t=2.219, df=13, 95% CI= 0.05 – 3.71, d=1.22)	350	**$p=0.011^{*}$** (t=2.95, df=13, 95% CI= 179.33-1167.45, d=1.61)	8.88	**$p=0.001^{£}$** (W=1)
**First dyad (9)**	174.6		4.82		781		22.36	
**Second or third dyad (6)**	212.7	$p=0.261^{*}$ (t=-1.18, df=13, 95% CI= -108.09 – 31.87, d=0.62)	5.9	$p=0.257^{*}$ (t=-1.19, df=13, 95% CI= -3.04 – 0.26, d=0.62)	827	$p=0.872^{*}$ (t=-0.16, df=13, 95% CI= -659.66-566.67, d=0.09)	22.58	$p=0.529^{£}$ (W=21)

*Repeat’ refers to dyads where the demonstrator was used in multiple dyads, and non-repeat those who were used just once. ‘First dyad’ refers to dyads where the demonstrator was being used for the first time, and ‘second or third dyad’ refers to dyads where the demonstrator had been used previously. Data were analysed with *unpaired one-sided t-tests or £two-tailed Mann-Whitney U tests, depending on the number of groups and the distributions of the data, with 95% confidence intervals (CI) and effect sizes presented as appropriate. Effect sizes for parametric tests were calculated using Cohen’s d for t-tests. Significant comparisons are marked in bold. To account for differences in session number, the demonstrator box-opening index was calculated as the total incidence of box opening by the demonstrator / number of joint foraging sessions. Following indexes were calculated as the total duration of following behaviour / number of joint foraging sessions. Following behaviour was defined as the observer being present on the surface of the box, within a bees’ length of the demonstrator, while the demonstrator performed box opening (thus, following behaviour could only occur after the demonstrator began pushing the blue tab and before it accessed the reward). These figures represent the average for the group. See Table 1 for individual demonstrator box-opening data, and Supplementary Table 1 for individual observer following data.*
