# Supplementary material for: Bumblebees socially learn behaviour too complex to innovate alone
Source: Nature. 2024 Mar 6;627(8004):572–8. doi: 10.1038/s41586-024-07126-4 (PMC10954542; doi:10.1038/s41586-024-07126-4)
Supplement: Supplementary file 2 — Reporting Summary [file 41586_2024_7126_MOESM2_ESM.pdf]

nature portfolio

Corresponding author(s): Alice D. Bridges

Last updated by author(s): Jan 16, 2024

Reporting Summary

Nature Portfolio wishes to improve the reproducibility of the work that we publish. This form provides structure for consistency and transparency in reporting. For further information on Nature Portfolio policies, see our Editorial Policies and the Editorial Policy Checklist.

Statistics

For all statistical analyses, confirm that the following items are present in the figure legend, table legend, main text, or Methods section.

n/a	Confirmed	
☐	☒	The exact sample size (n) for each experimental group/condition, given as a discrete number and unit of measurement
☐	☒	A statement on whether measurements were taken from distinct samples or whether the same sample was measured repeatedly
☐	☒	The statistical test(s) used AND whether they are one- or two-sided<br>*Only common tests should be described solely by name; describe more complex techniques in the Methods section.*
☒	☐	A description of all covariates tested
☐	☒	A description of any assumptions or corrections, such as tests of normality and adjustment for multiple comparisons
☐	☒	A full description of the statistical parameters including central tendency (e.g. means) or other basic estimates (e.g. regression coefficient) AND variation (e.g. standard deviation) or associated estimates of uncertainty (e.g. confidence intervals)
☐	☒	For null hypothesis testing, the test statistic (e.g. F, t, r) with confidence intervals, effect sizes, degrees of freedom and P value noted<br>*Give P values as exact values whenever suitable.*
☒	☐	For Bayesian analysis, information on the choice of priors and Markov chain Monte Carlo settings
☒	☐	For hierarchical and complex designs, identification of the appropriate level for tests and full reporting of outcomes
☐	☒	Estimates of effect sizes (e.g. Cohen's d, Pearson's r), indicating how they were calculated

Our web collection on statistics for biologists contains articles on many of the points above.

Software and code

Policy information about availability of computer code

Data collection	Data was collected from all videos using BORIS 7.10.2.
Data analysis	Data were analysed using R 4.0.4.

For manuscripts utilizing custom algorithms or software that are central to the research but not yet described in published literature, software must be made available to editors and reviewers. We strongly encourage code deposition in a community repository (e.g. GitHub). See the Nature Portfolio guidelines for submitting code & software for further information.

Data

Policy information about availability of data

All manuscripts must include a data availability statement. This statement should provide the following information, where applicable:

- Accession codes, unique identifiers, or web links for publicly available datasets
- A description of any restrictions on data availability
- For clinical datasets or third party data, please ensure that the statement adheres to our policy

Data underlying the figures presented in the manuscript are available on figshare: https://doi.org/10.6084/m9.figshare.24072486

Research involving human participants, their data, or biological material

Policy information about studies with human participants or human data. See also policy information about sex, gender (identity/presentation), and sexual orientation and race, ethnicity and racism.

Reporting on sex and gender	Not applicable
Reporting on race, ethnicity, or other socially relevant groupings	Not applicable
Population characteristics	Not applicable
Recruitment	Not applicable
Ethics oversight	Not applicable

Note that full information on the approval of the study protocol must also be provided in the manuscript.

Field-specific reporting

Please select the one below that is the best fit for your research. If you are not sure, read the appropriate sections before making your selection.

☒ Life sciences ☐ Behavioural & social sciences ☐ Ecological, evolutionary & environmental sciences

For a reference copy of the document with all sections, see nature.com/documents/nr-reporting-summary-flat.pdf

Life sciences study design

All studies must disclose on these points even when the disclosure is negative.

Sample size	Our sample size included 15 dyads, each including a trained demonstrator and a naive observer, and the dyads were split into groups for further exploratory analyses based on two factors: the outcome of the observer's learning test (pass vs. fail) and the demonstrator's preferred behavioural technique (squeezing vs. staggered-pushing). Our video analysis meant that we were able to track the behaviour of both the demonstrator and the observer throughout the entirety of the experiment, which included 30-40 joint foraging sessions, and we were able to extract detailed behavioural data from each foraging session for analysis. Our sample size selection was based on those used in other comparable studies.
Data exclusions	No data were excluded from the analysis.
Replication	Dyadic and 12-day control population behavioural assays were performed by two independent MSc students (A. Royka and T. Wilson). The 24-day control population experiment was performed by two research assistants (C. Lockwood and J. Richter). A total of 15 paired dyads (one trained demonstrator paired with one untrained observer) were tested, and two colonies of bees were exposed independently to the test stimuli for the control population experiments. Replication attempts for the control population experiments were all successful. For the 15 dyads, n=5 resulted in a successful learner, with n=10 observers failing to learn the task. We did not anticipate that all bees should learn this task, with non-learning as valid an outcome as learning. Interindividual variation in difficult cognitive tasks is normal and to be expected.
Randomization	For analysis of the dyad experiments, observers were split into three groups (squeezing-pass, squeezing-fail and staggered-fail) based on two conditions : whether they passed or failed an unrewarded learning test, and the predominant behavioural variant used by their paired demonstrator during the joint foraging sessions (squeezing vs. staggered-pushing). Thus, randomization was not applicable.
Blinding	This was an observational study of animal behaviour, and so did not require blinding.

Reporting for specific materials, systems and methods

We require information from authors about some types of materials, experimental systems and methods used in many studies. Here, indicate whether each material, system or method listed is relevant to your study. If you are not sure if a list item applies to your research, read the appropriate section before selecting a response.

Materials & experimental systems

n/a	Involved in the study
☒	☐ Antibodies
☒	☐ Eukaryotic cell lines
☒	☐ Palaeontology and archaeology
☐	☒ Animals and other organisms
☒	☐ Clinical data
☒	☐ Dual use research of concern
☒	☐ Plants

Methods

n/a	Involved in the study
☒	☐ ChIP-seq
☒	☐ Flow cytometry
☒	☐ MRI-based neuroimaging

Animals and other research organisms

Policy information about studies involving animals; ARRIVE guidelines recommended for reporting animal research, and Sex and Gender in Research

Laboratory animals	Bumblebees (Bombus terrestris ssp. audax). The age of the bees used in this study was typical for foragers in this species according to the literature, and in our study the specific age was not determined.
Wild animals	The study did not involve wild animals.
Reporting on sex	Only female bumblebees were included in this study, which involved foraging behaviour by worker bees. All workers are female, and males (drones) do not forage for the colony.
Field-collected samples	The study did not involve samples collected from the field.
Ethics oversight	No ethical approval is necessary for work on arthropods.

Note that full information on the approval of the study protocol must also be provided in the manuscript.

Plants

Seed stocks	Not applicable.
Novel plant genotypes	Not applicable.
Authentication	Not applicable.
